# A cross-sectional validation study of the verran and snyder-halpern sleep scale and the factors influencing sleep questionnaire in Dutch hospitals

**DOI:** 10.1371/journal.pone.0354633

**Published:** 2026-09-01

**Authors:** Christel T. A. J. Derks, Jessica J. J. Cramer-Kruit, Paul Lodder, RENurse Consortium, Annemarie J. B. M. de Vos, Catharina J. van Oostveen

**Affiliations:** 1 Elisabeth-TweeSteden Hospital, Hilvarenbeekse Weg 60, Tilburg, The Netherlands; 2 Martini Hospital, Van Swietenplein 1, Groningen, The Netherlands; 3 Department of Methodology & Statistics, Tilburg University, Tilburg, The Netherlands; 4 Fontys University of Applied Sciences, Professor Goossenslaan 1-01, Tilburg, The Netherlands; 5 Avans University of Applied Sciences, Hogeschoollaan 1, Breda, The Netherlands; 6 Spaarne Gasthuis Hospital, Spaarne Gasthuis Academy, Haarlem, The Netherlands; 7 Erasmus School of Health Policy & Management, Erasmus University Rotterdam Campus Woudestein, Rotterdam, The Netherlands; Universiti Sains Malaysia - Kampus Kesihatan, MALAYSIA

## Abstract

**Background:**

Adequate sleep is important for hospitalised patients, yet often disrupted by poor sleep hygiene. Limited research exists on sleep quality and contributing factors in general hospital wards, and reliable assessment tools are lacking. This study aimed to cross-culturally validate the Verran and Snyder-Halpern Sleep Scale (VSH) and the Factors Influencing Sleep Questionnaire (FISQ), assessing their psychometric properties among Dutch hospital patients, and evaluate patients sleep quality in general wards.

**Methods:**

A cross-sectional validation study included patients from internal medicine and surgical wards in six Dutch teaching hospitals. The VSH and the FISQ underwent a forward-backward translation process and were validated following COSMIN guidelines, including Confirmatory Factor Analysis (CFA). Sleep quality and hygiene were measured with the VSH (visual analogue scale) and FISQ (5-point Likert scale). Associations between patient characteristics and sleep hygiene factors were analysed using regression analyses.

**Results:**

A total of 693 respondents completed the questionnaire. Over half were aged 50−74 years and stayed in multi-occupancy rooms for more than four nights. Most (51%; n = 354) reported poorer sleep than at home. Reported disturbances included uncomfortable beds, pain, noisy ventilation, patient noise and equipment alarms. CFA revealed a three-factor model for the VSH and a one-factor model for the FISQ. Regression analysis showed daytime sleep was linked to more disturbance (β = 0,13[0,03, 0,23]) and lower sleep effectiveness (β = −0,11[−0,19, −0,03]). Fewer sleep hours were associated with lower sleep effectiveness (β = 0,57[0,48, 0,66]) and increased disturbance (β = −0,59[−0,67, −0,5]). More frequent sleep interruptions were associated with higher sleep disturbance (β = −0,22[−0,3, −0,15]) and increased need for sleep supplementation (β = −0,11[−0,21, 0]).

**Conclusions:**

The Dutch VSH and FISQ are valid, reliable tools for assessing sleep quality and hygiene. Despite measured suboptimall sleep conditions, these tools offer valuable insights for targeted improvements in hospital sleep hygiene.

## Background

Good sleep quality affects patients’ health as well as their recovery. It increases immunity, reduces depression and decreases demand for painkillers [1]. In addition, a good night’s sleep has a positive effect on cognitive and emotional functioning, well-being, and quality of life [2,3]. On the other hand, sleep deprivation has numerous negative cognitive, autonomic metabolic and hormonal effects on the body, including elevation of blood pressure and glucose levels [4–7]. These effects lead to delayed recovery, increased morbidity, including diabetes, coronary artery disease and delirium, and increased mortality [8–10].

Poor sleep quality is frequently observed in hospitalised patients [7,11]. Sleep is increasingly recognized as a fundamental component of health, as emphasized by the American Academy of Sleep Medicine (AASM), which identifies sleep as a biological necessity and warns that insufficient or poor‑quality sleep has significant physical, cognitive, and safety consequences [12]. The reasons for poor sleep quality are multifactorial and comprise patient-related factors, including pain and discomfort, and hospital environmental factors, including other patient’s noises and early ward rounds [7,13]. Nurses, who often overestimate both the quality of their patients’ sleep and their own contribution to sleep disturbance, also need to recognise their role in hospital sleep hygiene [14,15]. A Dutch multicentre study demonstrated that hospitalised patients’ sleep time is reduced by 83 minutes compared to at-home sleep time due to various hospital-related factors [16].

Disruptions in sleep patterns can be attributed to various factors within a hospital setting. These include, but are not limited to, noise generated by hospital staff, nocturnal awakenings instigated by nursing personnel, and environmental factors such as the operation of equipment or external traffic. Such disruptions have been identified as having detrimental impact on patient recovery processes, consequently extending the duration of hospital stay [13,16,17]. These disruptive elements are encompassed within the concept of sleep hygiene, a term that refers to the behaviours, practices, and environmental conditions that influence both the perceived and actual quality and quantity of sleep [18].

Various studies have shown that many sleep interruptions occur in patients admitted to an Intensive Care Unit [19–21]. However, there is a lack of knowledge regarding the relationship between sleep quality and its causes in general hospital wards [13,15].

The Verran and Snyder-Halpern Sleep Scale (VSH) and the Factors Influencing Sleep Questionnaire (FISQ) respectively measure sleep quality and sleep hygiene [22,23]. The VSH was developed to assess the subjective sleep quality of hospitalised individuals without pre-existing sleep difficulties. The FISQ measures the sleep disrupting factors. Both instruments proved sufficient validity and reliability in previous studies [22–27]. While poor sleep quality is common in hospitalized patients and negatively impacts recovery, there is limited understanding of the factors influencing sleep quality and hygiene in general hospital wards. The AASM also calls for greater attention to sleep health in clinical practice and recommends optimizing sleep conditions in inpatient settings. This underscores the importance of studying hospital sleep quality and the need for validated tools to assess sleep hygiene in clinical environments [12]. Existing tools to assess sleep quality, such as the Verran and Snyder-Halpern Sleep Scale (VSH) and the Factors Influencing Sleep Questionnaire (FISQ), have not been cross-culturally validated for use in Dutch hospital populations.

Therefore, the primary aim of this study is to cross-culturally validate the VSH and the FISQ and to evaluate their psychometric properties among Dutch hospital patients.

A secondary, exploratory aim is to examine sleep hygiene and to investigate the associations between patient characteristics and both sleep quality and sleep hygiene outcomes.

It is expected that the results of this study can be used to inform hospital policy regarding to staff- and environment-related factors to enhance sleep hygiene and ultimately improve patients’ sleep quality.

## Methods

### Study design

A cross-sectional study design was employed to evaluate sleep hygiene. The validation process for both the VSH and the FISQ adhered to the criteria set forth by the COnsensus-based Standards for the selection of health Measurement INstruments (COSMIN) [28]. The findings were reported in accordance with the guidelines specified in the Strengthening the Reporting of Observational Studies in Epidemiology (STROBE) statement [29] (S1 File).

### Study setting

The study sample was comprised of patients enlisted from six teaching hospitals situated in various regions of the Netherlands, encompassing both metropolitan and countryside areas. These hospitals are associated with the Research and Education in Nursing (RENurse) consortium. The consortium, see renurse.nl, represents a cooperative effort among fourteen Dutch Top Clinical hospitals. The primary objective of this collaboration is to enhance the quality of nursing care. This is achieved through the execution of scientific research, the dissemination of acquired knowledge, and the practical application of this knowledge in nursing practice.

### Respondent selection and recruitment

Each hospital had a consortium representative serving as the coordinator, who was tasked with the selection of a minimum of two general internal medicine wards and two surgical wards within their respective hospital. The coordinator secured permission from the department head to execute the study and established a point of contact for each ward. The hospital coordinator was charged with liaising with the wards and overseeing the research process within their hospital. By prior arrangement, the researchers delivered the printed questionnaires with informed letter and informed consent to the hospital, entrusting them to the hospital coordinator for distribution among the participating wards. The ward coordinator engaged potential respondents the second day the patients were admitted to the hospital. Patients were deemed eligible to participate if they met the following criteria: 1) proficient in Dutch, 2) cognitively competent, 3) admitted to the hospital for a minimum of one night, and 4) aged 18 or older. Data collection took place between 1 August and 22 December 2021.

### Sample size

Sample size was determined based on the primary aim of cross-cultural validation of the VSH and the FISQ, requiring adequate sample size for confirmatory factor analysis (CFA). The study was not powered based on precision of individual item responses, but on stable estimation of latent factor structures.

Following guidance by Wolf et al. (2013), traditional rules of thumb suggest approximately 10 respondents per indicator, corresponding to about 150 participants for the FISQ and 280 respondents for the VSH. Simulation studies further indicate that required sample sizes for CFA vary depending on model complexity and factor loadings [30]. Therefore, a conservative target sample size of approximately 450 respondents across six hospitals was used to ensure sufficient power for robust psychometric evaluation.

### Instruments

The VSH was selected due to its multidimensional assessment of sleep in hospitalised patients and its suitability for capturing acute in-hospital sleep experiences. Compared with general sleep instruments such as the Pittsburgh Sleep Quality Index and the Insomnia Severity Index, which primarily assess chronic or general sleep problems, the VSH is specifically designed for short- term, hospital-based measurement and is sensitive to environmental sleep disruption inpatient settings [31–33]. The Richards–Campbell Sleep Questionnaire was considered but not selected due to its focus on perceived sleep quality in intensive care populations, limiting its applicability to general hospital wards [34–36]. The Potential Hospital Sleep Disruptions and Noises Questionnaire (PHSDNQ) was also considered; however, this instrument focuses primarily on environmental noise and does not capture broader multidimensional sleep of sleep quality relevant to the present study [37].

The FISQ was included to assess environmental, clinical, and patient-related factors influencing sleep that are not captured by the VSH, thereby providing complementary information on modifiable determinants of sleep disruption in hospital settings.

### Verran and Snyder-Halpern Sleep Scale

The Verran and Snyder-Halpern Sleep Scale (VSH) was developed by Verran and Snyder-Halpern in 1990 as a subjective instrument for assessing sleep characteristics. The VSH evaluates three dimensions of sleep: sleep disturbance, sleep effectiveness, and sleep supplementation. The VSH comprises fourteen open-ended questions regarding perceived sleep quality and habits before bedtime, and fifteen items that assess the characteristics of the previous night’s sleep, scored on a visual analogue scale [27]. Each of these fifteen items features a visual analogue response on a 10 cm line, with endpoints verbally described, representing both extremes of the scale, and numerically ranging from 0 to 100. A higher aggregate score indicates superior sleep quality. The maximum score for the sleep disturbance dimension is 700 (7 items), for sleep effectiveness 400 (4 items), and for sleep supplementation 400 (4 items). The Cronbach’s Alpha coefficient demonstrated an estimated reliability of 0.82 [23].

### Factors influencing sleep questionnaire

The Factors Influencing Sleep Questionnaire (FISQ) quantifies the number and intensity of elements that patients perceive as detrimental to their sleep. The FISQ was initially developed for patients admitted to clinical wards, comprising 35 items and four constructs [22].

In this study, 28 items of the original FISQ were retained. Seven items (‘comfort’, ‘procedures on patients’, ‘routine before sleep’, ‘equipment’, ‘others’ interruptions’, ‘doctors’ interruptions’, and ‘traffic outside hospital’) were excluded prior to data collection as part of the cross-cultural adaption process. These items were considered either not sufficiently specific, open to multiple interpretations (e.g., ‘comfort’ and bedtime routine’), or less applicable to the current hospital contexts (e.g.,’ traffic outside the hospital’ due to low-traffic locations of the participating hospitals). Additionally, some items were considered difficult for hospitalised patients to assess consistently.

The selection of items was guided by considerations of relevance and feasibility for the study population. To support the content validity and clarity of the adapted instrument, the translated and adapted version of the FISQ was reviewed by an expert panel consisting of healthcare professionals with expertise in geriatric medicine, nursing, and patient care. Together with the research team, the panel evaluated the relevance, clarity and applicability of the items and reached consensus regarding the removal of the seven items described above. This process helped to evaluate the relevance, clarity and applicability of the included items within the Dutch hospital setting.

Respondents evaluated each item on a 5-point Likert scale (0 = ‘not at all’; 1 = ‘somewhat’; 2 = ‘moderate’; 3 = ‘quite a bit’; 4 = ‘extremely’). Items scoring ‘moderate’, ‘quite a bit’, or ‘extremely’ were considered sleep-disrupting. The overall score was calculated by summing item scores of the 28 items and dividing by the number of items, resulting in an average score representing perceived sleep hygiene [22,26]. The FISQ exhibited satisfactory content validity and internal consistency in a study of sleep in postoperative patients (Cronbach’s Alpha = 0.94) [22].

### Translation process

The translation process of the VSH and FISQ from British English into Dutch was conducted in accordance with the guidelines proposed by Wild et al 2005 [38]. First, three independent bilingual individuals translated the VSH and FISQ from English to Dutch, according to provided instructions. Emphasis was placed on conceptual rather than verbatim translation. The backward translation was executed by two independent translators whose mother tongue is English and who have no knowledge of the questionnaire or medical background. The final step in the translation process was to identify and resolve differences, as well as inadequate expressions or concepts in the translated version by an expert panel, consisting of one internist of geriatric medicine, one gerontologist, one nurse specialist neurologic and two nurses. This resulted in a final Dutch version of the VSH and FISQ (Supplemental File 2). A side-by-side overview of the original English items and their Dutch translations is provided in S3 File.

### Data analysis

For the purpose of cross-cultural validation, Confirmatory Factor Analysis (CFA) was executed to as the factorial and convergent validity of the VSH and the FISQ. Some questionnaire items contained missing responses (approximately 5–10% per item for the FISQ and 10–15% for the VSH). Higher missingness was observed for VSH items 11–13, likely due to their reversed wording, suggesting a Missing At Random (MAR) mechanism related to item characteristics. Missing data in the VSH CFA were handled using full information maximum likelihood (FIML), which provides valid estimates under MAR. For the categorical FISQ CFA, weighted least squares estimation with mean and variance adjustment (WLSMV) was used, based on pairwise available data. Given the relatively low proportion of missing data, this approach was considered appropriate.

Next, a latent regression analysis within the Structural Equation Modeling framework was performed to discern the relationship between patient characteristics and sleep hygiene factors. The demographic data comprised both nominal and continuous variables. Frequencies and percentages were computed for the nominal data. Means and standard deviations were calculated for the normally distributed continuous variables, while median and interquartile range were calculated for the non-normally distributed continuous variables.

### Confirmatory factor analysis

The CFA was used to test the originally proposed 3-factor structure for the VSH and the 4-factor structure for the FISQ, in comparison with 1-factor models. A categorical CFA with Weighted Least Squares (WLS) estimation was utilized to model the ordinal FISQ item scores, while a linear CFA with Robust Maximum Likelihood (MLR) estimation was employed to model the continuous VSH item scores. A model was deemed to adequately fit the data if at least two of the following criteria were met: a Comparative Fit Index (CFI) greater than 0.95; a Standardized Root Mean Square Residual (SRMR) less than 0.08; and a Root Mean Square Error of Approximation (RMSEA) less than 0.06 [39–41]. In case of misfit, modification indices were inspected. Residual item covariances were added if this could significantly enhance model fit. The reliability was investigated by separately estimating the McDonald’s omega based on the best fitting model of the FISQ and VSH.

### Latent regression analysis

A latent regression analysis was conducted within the framework of the structural equation modelling to ascertain the correlation between patient characteristics and factors of sleep hygiene. The most suitable CFA model for both VSH and FISQ was expanded to include the following predictors of sleep quality and sleep hygiene factor scores: ‘gender’, ‘age’, ‘type of admission’ (acute or planned), ‘room occupancy’ (single or multiple), ‘number of nights included’, ‘quality of sleep’, ‘duration of sleep at home or hospital’ (both during the day and at night), and ‘sleep interruptions’. Due to the extensive number of relationships expressed in β’s and CI’s, a Bonferroni correction was employed to adjust the level of significance. For all other analyses, a significance level of 0.05 was deemed indicative of statistical significance. The dataset used for statistical analyses is provided as Supporting information (S7 File). The CFA, latent regression analysis and reliability analysis were conducted using R (R version 3.6.0 and R studio version 1.2.1335). All other analyses were carried out using the Statistical Packages for the Social Sciences (SPSS), version 28.0.

### Ethics considerations

The study was reviewed and issued a non-WMO declaration by the Medical Research Ethics Committee Brabant, The Netherlands (NW2021−65). All participating hospitals provided local approval to conduct the study in accordance with their institutional research governance procedures. Prior to enrolment, respondents. Prior to enrolment, respondents received written study information and an informed consent form together with the questionnaire. Informed consent was obtained prior to participation. Completion and return of the questionnaire, together with the signed consent form, was taken as documentation of informed consent.

## Results

### Characteristics

In total, 771 questionnaires were received. Of these, 62 respondents did not meet the inclusion criteria, twelve did not provide informed consent, and four provided consent but did not complete the questionnaire. This resulted in a final sample of 693 respondents. The respondents (n = 693) were mostly men (51.8%) and between 50–74 years old. Most respondents (67.2%) were acutely admitted and stayed in a multi-occupancy room (70.7%). Respondents’ average length of stay, measured in nights, was 4 nights (median = 3) (Table 1).

**Table 1:** Characteristics of respondents.

	N _(total = 693)_ (%)
**Sex**	
Male	359 (51.8)
Female	328 (47.3)
Other	1 (0.1)
**Age (years)**	
≤24	21 (0.3)
25-49	88 (12.7)
50-74	389 (56.1)
≥75	189 (27.3)
**Admission**	
Acute	466 (67.2)
Elective	225 (32.5)
**Room**	
Single-occupancy room	196 (28.3)
Multi-occupancy room	490 (70.7)
Length of stay (nights), median (IQR)	3 (1-5)

*Percentages do not add up to 100% due to rounding

### Validation of VSH and FISQ

The CFA indicated that the original three-factor model of the VSH did not fit the data well (Table 2). After calculating the model fit, the modification indices suggested that model fit could be improved by adding residual correlations between similar items (S4 File). Eight residual correlations were added step by step, resulting in an adjusted three-factor model that showed good model fit (Table 2). The final adjusted three-factor model is presented in S6 File. Factor loadings and reliability estimates for the final model are provided in S5 File. For the D-VSH, reliability estimated by McDonald’s omega was 0.80 for sleep disturbance, 0.74 for sleep effectiveness, 0.63 for sleep supplementation, and 0.86 for the total score. While omega values for sleep disturbance and effectiveness indicated acceptable internal consistency, the lower omega for sleep supplementation suggests limited internal consistency of this subscale.

**Table 2:** VSH and FISQ; fit of the original and final model.

	Model	Normed χ 2	CFI	SRMR	RMSEA
VSH	3-factor	418.1*	0.879	**0.068**	0.084
	3-factor adjusted	217.1*	**0.951**	**0.059**	**0.056**
FISQ	1-factor	1364.3*	**0.975**	0.081	0.065
	1-factor adjusted	1192.3*	**0.981**	**0.078**	**0.057**

Bold cells indicate acceptable fit * *p* < .05

The original four-factor model for the FISQ did not converge because the covariance matrix of the four latent variables was not positive definite. Specifically, several Heywood cases were observed with latent factor correlations exceeded 1.0, indicating that the factors were not empirically distinguishable in our sample. Given this extreme collinearity among the latent factors, the four-factor solution did not provide a tenable representation of the data. In contrast, a more parsimonious one-factor model showed good fit and yielded a well-behaved solution without estimation problems. Therefore, a one-factor model was fitted to the data, which showed acceptable fit based on the SRMR (.081) and RMSEA (.065). Slightly better fit was achieved by adding one residual correlation between items 2 and 3 (‘comfort bed environment’ and ‘air-conditioning/ heating/ventilation’), yet given the minor improvement we decided to retain the initial one-factor model for reasons of parsimony. This resulted in a final one-factor structure for the D-FISQ (Table 2). Factor loadings estimate for the D-FISQ are provided in S5 File. Reliability analysis indicated excellent reliability for the D-FISQ with an estimated McDonald’s omega of 0.94.

### Sleep quality

The majority of the respondents (51%) reported to sleep less hours in the hospital compared to at home. On average, respondents slept 7.5 hours at home during the night and 6 hours at the hospital (Table 3). In addition, 49.4% (n = 342) respondents reported napping during daytime. One hundred thirty-one respondents (20.8%) reported to have one or more rituals before going to sleep. These rituals were bathroom related (n = 77), an activity (n = 41), intake of medication (n = 13), spiritual nature related (n = 11), eating or drinking (n = 4), preparing surroundings (n = 4), stopping an activity (n = 3), physical contact with family members (n = 2) or a combination of these rituals.

**Table 3:** Sleep quality and sleep hygiene; VSH and FISQ scores.

	Mean (SD)
Hours of sleep at home	7.17 (1.33)
Hours of sleep at hospital	5.56 (2.02)
VSH dimensions	
Sleep disturbance (700*)	385.33
Sleep effectiveness (400*)	198.22
Sleep supplementation (400*)	288.26
FISQ	13.58 (13.72)
Rituals before bed (N)	131

*Maximum score

### Sleep disturbance factors

Respondents scored an average of 13.58 (SD 13.72) on the FISQ (Table 3). The five most disturbance factors were ’pain’, ‘comfort of bed and surroundings’, ‘ventilation system’, ‘equipment alarms’ and ‘patient sounds’ (Table 4).

**Table 4:** Sleep disturbance factors.

	Median (IQR)
**Pain**	**1 (0-3)**
**Comfort of bed and surroundings**	**2 (0-3)**
**Ventilation system**	**1 (0-2)**
Anxiety	0 (0-0)
Nurses’ interruptions	0 (0-1)
Lights	0 (0-1)
Doors opening/closing/slamming	0 (0-1)
Talking at nurses’ station	0 (0-0)
**Equipment alarms**	**1 (0-1)**
Paging	0 (0-0)
Talking in hallway at night	0 (0-0)
Visitors	0 (0-0)
Talking in hallway during day	0 (0-1)
Cleaning equipment	0 (0-0)
Footsteps	0 (0-1)
Handwashing	0 (0-0)
Intercom	0 (0-0)
Staff talking at bedside	0 (0-1)
Squeaky equipment	0 (0-1)
**Patient sounds**	**1 (0-2)**
Television	0 (0-0)
Toilet flushing	0 (0-0)
Procedures on other patients	0 (0-0)
Falling objects	0 (0-0)
Telephone	0 (0-0)
Trolleys in hallway	0 (0-0)
Radio	0 (0-0)
Meal trays	0 (0-0)

Note: *omega total reliability estimate = 0.93*

### Relationship between respondent characteristics and sleep quality and sleep hygiene

A latent regression analysis was used to estimate the relation between each of the sleep hygiene latent variables as dependent variable and various patient characteristics as independent variables. The variance explained by the models is respectively 12.5% for FISQ, 39.9% for VSH sleep effectiveness, 48.3% for VSH sleep disturbance and 23.5% for VSH sleep supplementation. Respondents admitted to a multi-occupancy room (β = 0.15 [0.06, 0.25]) and respondents who reported low sleep quality during their hospital stay and (β = −0.16 [−0.27, −0.05]), scored significantly higher on the FISQ. Respondents who reported taking naps during daytime also had higher scores on the FISQ (β = 0.13 [0.03, 0.23]). Respondents who reported sleeping during the day experienced a greater number of disturbance factors on the FISQ and demonstrated lower sleep effectiveness (β = −0,11[−0.19, −0.03]). Respondents who reported less hours of sleep in the hospital experienced lower sleep effectiveness 0,57 [0.48, 0.66] and more sleep disturbance (β = −0.59 [−0.67, −0.5]) according to the VSH. Additionally, respondents experiencing more frequent sleep interruptions exhibited higher scores for sleep disturbance (β = −0.22 [−0.3, −0.15]) and sleep supplementation (β = −0.11 [−0.21, 0]) (Table 5).

**Table 5:** Relationship between respondent characteristics, sleep quality and sleep hygiene factors according to four latent regression analyses.

Patient characteristics	FISQ β [95% CI]	VSH Sleep effectiveness β [95% CI]	VSH Sleep disturbance β [95%CI]	VSH Sleep supplementation β [95% CI]
Gender	0.04 [−0.05, 0.13]	−0.09 [−0.16, −0.01]*	0.07 [0, 0.15]*	0.06 [−0.05, 0.16]
Age	−0.09 [−0.18, 0]	0.02 [−0.06, 0.1]	−0.03 [−0.1, 0.04]	−0.05 [−0.15, 0.06]
Admitted acutely or planned	−0.03 [−0.12, 0.07]	0.07 [−0.01, 0.15]	−0.04 [−0.11, 0.04]	0.1 [0, 0.21]*
Single or multi-occupancy room	0.15 [0.06, 0.25]*	−0.03 [−0.11, 0.05]	0.06 [−0.02, 0.13]	0.08 [−0.02, 0.18]
Number of nights in hospital	0.11 [0.01, 0.21]*	0.06 [−0.02, 0.13]	−0.06 [−0.14, 0.01]	0.05 [−0.05, 0.16]
Sleep quality	−0.16 [−0.27, −0.05]*	0.08 [−0.02, 0.17]	−0.09 [−0.18, 0]	0.08 [−0.04, 0.21]
Hours of sleep at home	0.04 [−0.08, 0.15]	−0.03 [−0.12, 0.06]	−0.01 [−0.09, 0.08]	0.02 [−0.1, 0.14]
Hours of sleep in the hospital	−0.14 [−0.26, −0.01]*	0.57 [0.48, 0.66]*	−0.59 [−0.67, −0.5]*	−0.01 [−0.15, 0.12]
Hours of sleep at home during the day	−0.02 [−0.13, 0.08]	0.05 [−0.04, 0.13]	−0.01 [−0.09, 0.07]	0.04 [−0.07, 0.15]
Hours of sleep in the hospital during the day	0.13 [0.03, 0.23]*	−0.11 [−0.19, −0.03]*	0.01 [−0.07, 0.09]	0.42 [0.32, 0.53]
Sleep interruptions	−0.02 [−0.11, 0.07]	0.1 [0.02, 0.18]*	−0.22 [−0.3, −0.15]*	−0.11 [−0.21, 0]*

* p < .05 Bold faced cells are significant after Bonferroni correction for multiple testing

## Discussion

This study conducted a cross-cultural validation of the VSH and FISQ and evaluated their psychometric properties in Dutch hospital patients. Both instruments demonstrated satisfactory validity and reliability for assessing sleep quality and sleep hygiene in hospitalized populations. These findings establish the first psychometrically validated Dutch versions of the VSH (D-VSH) and FISQ (D-FISQ), supporting structured assessment of sleep and related factors in Dutch clinical practice.

The D-VSH retained all original items and demonstrated a three-factor structure, sleep disturbance, sleep effectiveness, and sleep supplementation, that showed good model fit and aligns with the theoretical structure of the original scale [23,27]. This supports the structural stability of the VSH across cultural contexts. In contrast, the D-FISQ deviated from the original four-factor structure. The theoretically proposed model could not be retained due to statistical inadmissibility, as indicated by a non-positive definite covariance matrix and excessively high correlations between latent factors [22,26]. This suggests that the original dimensions are not empirically distinguishable in the hospital patient population.

Instead, a more parsimonious one-factor model demonstrated good fit and stable estimation. This indicates that, within the Dutch hospital context, the FISQ items may reflect a single overarching construct of sleep-disrupting factors rather than distinct domains. This finding may be explained by the high degree of overlap between environmental and care-related disturbances in contemporary hospital settings, where multiple factors co-occur and are experienced as a combined burden by patients. The reduction of seven items, due to limited relevance or interpretability, may have further contributed to this structural shift. Taken together, these results suggest that the conceptual distinction between different domains, such as environmental noise, care activities, and physiological factors, may be less meaningful from the patient perspective than originally assumed.

Both instruments demonstrated satisfactory internal consistency and test–retest reliability, although Cronbach’s alpha values were slightly lower than those reported for the original instruments [42]. This may reflect differences in population characteristics, translation nuances, or contextual factors related to the Dutch healthcare setting. The sleep supplementation subscale of the D-VSH showed modest reliability, which appears to be related to conceptual heterogeneity within the construct. The included items capture different aspects of sleep functioning, such as daytime sleepiness, difficulty waking, and the need for additional rest, which may not represent a unidimensional construct. This interpretation is supported by relatively low inter-item correlations and only a modest increase in reliability when using McDonald’s omega. These findings suggest that sleep supplementation may be better conceptualized as a multidimensional construct in future research.

Our findings regarding the psychometric properties of the D-VSH align with earlier validation studies in other cultural contexts. Mashayekhi et al. (2016) demonstrated acceptable reliability and construct validity of the Persian VSH, supporting its cross-cultural applicability [43]. Together, these findings indicate that the VSH is robust across languages and cultural settings. Our study extends this evidence by applying COSMIN guidelines and confirmatory factor analysis, providing stronger support for the structural validity of the Dutch version [22,26].

In terms of sleep outcomes respondents reported poorer sleep quality in the hospital compared to their home environment, with an average reduction of 1.5 sleep hours. This finding is consistent with international literature demonstrating that sleep disruption is highly prevalent in with previous research, that highlights frequent interruptions, earlier awakenings, and reduced sleep quality among hospitalized patients [15,16]. For example, the systematic review by Burger et al. (2022), which included 203 studies with 17.964 patients, reported similarly poorer sleep quality, with frequent nocturnal awakenings and an average of sleep duration of 5.7 hours [15].

The D-VSH identified several potential factors associated with ’sleep quality. Of the respondents, 131 reported having bedtime rituals, which varied significantly in nature. These routines were conceptualized as components of sleep hygiene. Assessing individual sleep habits may provide healthcare professionals with valuable insights into patients’ usual behaviors and preferences. Tailoring interventions to these routines may improve patient comfort during hospitalization, highlighting the importance of individualized, patient-centered approaches to sleep promotion [44].

In our study, patients in multi-patient occupancy rooms reported poorer sleep quality due to disruptions, consistent with previous studies [13,45,46]. Key disturbances, including noise, multi-occupancy rooms, pain, and anxiety, closely mirror those identified in our Dutch sample, thereby reinforcing the validity of our findings. Prior research also highlights frequent interruptions and early awakenings in hospital settings [15,16] with environmental factors, such as hospital infrastructure, contributing to this disturbance, as patients admitted to newer facilities reported better sleep outcomes [47]. Additionally, respondents reported increased daytime napping and the use of over-the counter sleep aids, such as low-dose melatonin or herbal supplements; however, increased daytime time napping may negatively affect nocturnal sleep quality [48–52]. Our findings support existing recommendation, as patients frequently reported sleep disturbance caused by nursing staff activities, such as administering medication or changing infusion fluids during the night, underscoring the need for targeted interventions. Such interventions may address issues related to suboptimal sleep hygiene. Finally, response to the question on shifts work were sparse, likely reflecting the demographic characteristics of the population, which primarily consisted of retired individuals or those not engaged in shift-based occupations, thereby limiting interpretation of this factor.

The broader clinical importance of these findings is emphasized by the AASM, which identifies sleep as a fundamental biological necessity [12]. Insufficient or poor sleep is associated with adverse physical, cognitive, and safety outcomes, and the AASM calls for greater attention to sleep health in clinical practice, including optimizing inpatient sleep conditions. Our findings demonstrating substantial sleep loss and exposure to environmental and care-related disturbances reinforce the need for systematic attention to sleep as an integral component of hospital care [12].

Recent evidence highlights that sleep disturbances in hospitalized patients arise from a complex interplay of environmental, medical, psychological, and physiological factors and are associated with adverse outcomes [53]. This work emphasizes the importance of a multidisciplinary approach and recommends prioritizing non-pharmacological interventions, like earplugs, eye masks, and music [54].

### Strengths and limitations

A key strength of this study is the inclusion of a substantial cohort of patients admitted to general hospitals across the Netherlands. This enhances the generalizability of the findings within the Dutch hospital context and provides a comprehensive overview of patient- reported sleep quality and sleep hygiene practices. The multicenter nature of the data collection further strengthens the ecological validity of the findings.

An additional strength is the rigor of the psychometric evaluation. This study employed CFA and assessed model stability across specifications, thereby strengthening confidence in the observed factor structure of the VSH. Sensitivity analyses showed that the inclusion of correlated residuals improved model fit but did not materially affect regression coefficients or statistical significance. This indicates that the substantive relationships between constructs remain stable across different model specifications, supporting the robustness of the findings despite potential local item dependencies.

Several limitations should also be acknowledged. First, patients aged 75 years and older were underrepresented in the sample. This likely reflects challenges in independent questionnaire completion due to physical or cognitive limitations. Nevertheless, it is unlikely that other factors affected sleep quality in patients aged 75 and older differ significantly from those identified in our study. Hence, this limitation is not expected to significantly influence the study findings.

Second, a moderate level of missing data was observed for the D-VSH, with higher rates for items 11–13. These items were more frequently left unanswered, likely due to reversed wording. This pattern suggests that missingness was related to item characteristics rather than the underlying construct, consistent with MAR mechanism conditional on item characteristics. The use of FIML for the VSH and weighted lest squares estimation for the FISQ allowed appropriate handling of missing data under these conditions. Given the relatively low proportion of missing data, the potential impact on parameter estimates is considered limited and unlikely to have influenced the substantive conclusions. Nevertheless, some uncertainty in factor loading and inter-item correlations cannot be fully excluded, and the CFA results should be interpreted with appropriate caution.

Third, acceptable model fit for the VSH three-factor structure depends partly on the inclusion of correlated residuals, which may indicate local item dependence or partial item redundancy. Although sensitivity analyses demonstrated that structural regression estimates were robust to model specification, this feature may limit the generalizability of the exact measurement model across different populations and should be considered when applying the instrument in future research.

Finally, data collection was conducted between two COVID-19 waves. While no direct impact of the pandemic on study outcomes is expected, changes in hospital routines or patient experiences during this period cannot be fully excluded.

Importantly, cross-linguistic measurement invariance was not assessed in this study. Therefore, it remains unclear whether the Dutch version of the instrument functions equivalently across cultural and linguistic contexts.

### Implications for practice and research

This study highlights that sleep quality in hospital wards is suboptimal. The validated instruments provide a valuable tool for evaluating the effectiveness of interventions aimed at enhancing sleep hygiene. Hence, the implications for clinical practice are substantial and span multiple levels within the hospital organization.

Implementing a hospital-wide, non-pharmacological sleep hygiene protocol is essential to improve patients’ sleep experiences. Such a protocol is critical for raising awareness of nursing practices related to sleep and minimizing disturbance to patients’ sleep [55]. By implementing this protocol, hospitals can emphasize the importance of sleep as a vital component of patient care, thereby encouraging nursing staff to adopt targeted work processes and use appropriate materials. Furthermore, it provides healthcare management and professionals with tools to systematically prioritize sleep hygiene within the organization.

To improve sleep quality at the ward level, specific interventions can be implemented. For example, patients could be informed during the admission interview about the possibility of bringing personal items, such as their own pillow or bedding, as poor bed comfort has been identified as a significant factor for disrupting sleep quality [13,46]. Previous research has shown that a different bed and an uncomfortable pillow are common reasons for reduced sleep quality in hospital [56]. Additionally, nurses should be attentive to environmental factors in patient rooms, particularly noise and lighting, as these are frequently reported as disruptive and have a substantial impact on patients’ sleep quality. Managing noise from medical equipment, ventilation systems, and other patients is especially important to minimize sleep disturbances.

In line with these findings, Fang et al. (2024) demonstrated that simple interventions such as earplugs, eye masks, and music can significantly improve sleep quality. Given that our study also identified environmental noise as a key disturbance, these low-cost interventions represent practical and scalable strategies to improve sleep hygiene in hospital settings. Providing earplugs can help reduce night-time noise discomfort, while eye masks offer an effective solution for excessive light exposure in patient rooms [13,16,57–59].

Lastly, although the current findings provide evidence regarding the internal structure and reliability of the Dutch version, it remains unclear whether the translated instrument functions equivalently across languages and cultural contexts. Future studies should therefore recruit bilingual participants to directly compare responses from participants completing the original and translated versions to examine configural, metric, and scalar invariance. Such analyses would help determine whether the Dutch adaptation measures the same latent construct in the same manner as the original instrument and whether scores can be meaningfully compared across cultural or linguistic groups.

## Conclusion

The D-VSH and D-FISQ are valid and reliable instruments for measuring sleep quality and sleep hygiene in patients admitted to general wards in Dutch hospitals. Patients experience disturbed sleep while admitted in hospital, due to pain, discomfort of the bed, noisy ventilation systems, equipment alarms, and patient sounds. The findings of this study provide guidance to adjust, policies and give professionals tools to implement non-pharmacological interventions, such as the implementation of a comprehensive sleep hygiene protocol.

## Supporting information

S1 File Supporting information (Strobe checklist). (DOCX)

S2 File Dutch VSH and FISQ questionnaire. (DOCX)

S3 File Translation English- Dutch VSH and FISQ. (DOCX)

S4 File Residual item covariances added to the VSH model. (DOCX)

S5 File Factor loadings and reliability estimates for the VSH and FISQ. (DOCX)

S6 File Adjusted 3-factor model of the VSH instrument. (TIF)

S7 File Anonymized dataset. (XLSX)
