# Supplementary material for: A cross-sectional validation study of the verran and snyder-halpern sleep scale and the factors influencing sleep questionnaire in Dutch hospitals
Source: PLoS One. 2026 Sep 1;21(9):e0354633. doi: 10.1371/journal.pone.0354633 (PMC13533380; doi:10.1371/journal.pone.0354633)
Supplement: Supplementary file 2 [file pone.0354633.s002.docx]

**Supplemental file 2: Dutch version of the Verran and Snyder-Halpern Sleep Scale (VSH) and the Factors Influencing Sleep Questionnaire (FISQ)**

## **D-VSH Schaal**

1. Had u, voordat u in het ziekenhuis werd opgenomen, al regelmatig problemen met slapen?

□ Ja	□ Nee

2. Is er ooit een slaapprobleem bij u vastgesteld?

□ Ja	□ Nee

3a. Heeft u in het afgelopen jaar medicatie ingenomen om te helpen bij het slapen?

□ Ja	□ Nee	→ Indien u Nee heeft aangekruist, kunt u meteen door vraag #4.

3b. Als u JA heeft geantwoord op vraag 3a, hoeveel nachten neemt u gemiddeld thuis slaapmedicatie?

……………………………………………………………………………………………………………

4. Heeft u in het afgelopen jaar in ploegendiensten gewerkt?

□ Ja	□ Nee

5a. Hoe is uw slaapkwaliteit in het ziekenhuis in vergelijking met elders (bijvoorbeeld thuis)?

□ Beter	□ Slechter	□ Gelijk → indien ‘’gelijk’’ ga naar vraag 6

5b. Wanneer u **BETER of SLECHTER** heeft geantwoord bij vraag 5a, waarom denkt u dat de kwaliteit van uw slaap in het ziekenhuis beter of slechter is?

……………………………………………………………………………………………………………

6a. Hoeveel uur slaapt u gemiddeld ‘s nachts thuis?

........... uur

6b. Hoeveel uur slaapt u gemiddeld per nacht in het ziekenhuis?

........... uur

7a. Hoeveel uur slaapt u gemiddeld overdag thuis(bijvoorbeeld dutjes)?

........... uur

7b. Hoeveel uur slaapt u gemiddeld overdag in het ziekenhuis (bijvoorbeeld dutjes)?

........... uur

8. Hoe laat viel u voor het eerst in slaap gisteravond?
……………………………………………………………………………………………………………

9. Hoe laat werd u vanmorgen wakker?

……………………………………………………………………………………………………………

10. Sliep u aan een stuk door?

□ Ja	□ Nee

**Hierna volgen een aantal voorbeeldvragen:**

*Zet een X op de plaats die het beste aangeeft hoe u afgelopen nacht geslapen heeft. Onderstaande voorbeelden geven aan hoe u de vragen dient te beantwoorden*

***Voorbeeld A:***

*1. Tijdens mijn slaap van de afgelopen nacht:*

*Werd ik niet wakker	Was ik 10 uur wakker*

*0**10*

*Als u meer dan 10 uur wakker was, dan zet u uw X aan het eind van de lijn, naast de woorden “Was ik 10 uur wakker.”*

***Voorbeeld B:***

*Tijdens mijn slaap van de afgelopen nacht:*

*Werd ik niet wakker	Was ik 10 uur wakker*

*0**10*

*Als u 5 uur wakker was, dan zet u uw X in het midden van de lijn.*

***Voorbeeld C:***

*Tijdens mijn slaap van de afgelopen nacht:*

*Werd ik niet wakker	Was ik 10 uur wakker*

*0**10*

*Als u de hele nacht geslapen heeft, dan zet u uw X aan het begin van de lijn.*

**Hier wordt de vragenlijst vervolgd:**

**Plaats de X op de plek op de antwoordlijn die volgens u het beste weergeeft hoe u de afgelopen nacht heeft geslapen.**

11. Ik werd niet wakker	Was ik 10 uur wakker

0 10

12. Heb ik niet geslapen	Heb ik 10 uur geslapen

0 10

13. Gisteren overdag heb ik geslapen	Gisteren heb ik 10 uur overdag geslapen

0 10

14. Ik heb niet geslapen gisterochtend	Ik heb gisterochtend zo nu en dan geslapen

0 10

15. Ik heb niet geslapen gisteravond	Ik heb gisteravond geslapen

0 10

16. Gisteravond viel ik onmiddellijk in slaap	Gisteravond viel niet in slaap

0 10

17. Licht geslapen	Diep geslapen

0 10

18. Geen last van verstoorde slaap	Veel last van verstoorde slaap

0 10

19. Werd ik niet wakker	Was ik afwisselend wakker gedurende de hele nacht

0 10

20. Had geen problemen met in slaap komen	Had veel problemen met in slaap komen

0 10

21. Niet bewogen	De hele nacht liggen draaien

0 10

22. Vanochtend werd ik uitgerust wakker	Werd ik uitgeput wakker

0 10

23. Na het ontwaken vanochtend ben ik wakker gebleven	Na het ontwaken vanochtend ben ik weer in slaap gedommeld

0 10

24. Een slechte nachtrust gehad	Een goede nachtrust gehad

0 10

25. Had voldoende slaap	Niet voldoende slaap

0 10

## **D-FISQ**

Onderstaande 29 factoren zouden mogelijk van invloed kunnen zijn op uw slaap. Kruis het vakje aan per factor wat op uw slaapkwaliteit van toepassing is. De categorieën lopen op van laag naar hoog.

| **Factoren van invloed op slaap** | **Helemaal niet** | **Enigszins** | **Matig** | **Behoorlijk** | **Enorm** |
| --- | --- | --- | --- | --- | --- |
| Pijn |  |  |  |  |  |
| Comfort van bed en omgeving |  |  |  |  |  |
| Airconditioning, verwarming of ventilatiesysteem |  |  |  |  |  |
| Angst |  |  |  |  |  |
| Verstoring door verpleegkundigen |  |  |  |  |  |
| Verlichting |  |  |  |  |  |
| Deuren die geopend, gesloten of dichtgeslagen worden |  |  |  |  |  |
| Gesprekken in personeelsruimte |  |  |  |  |  |
| Alarmen op apparatuur |  |  |  |  |  |
| Oproepsysteem |  |  |  |  |  |
| ’s Nachts luid gepraat in de gang |  |  |  |  |  |
| Bezoekers |  |  |  |  |  |
| Luid gepraat overdag |  |  |  |  |  |
| Schoonmaak apparatuur zoals stofzuiger |  |  |  |  |  |
| Voetstappen |  |  |  |  |  |
| Handen wassen bij een wasbak in de buurt |  |  |  |  |  |
| Intercom |  |  |  |  |  |
| Gesprekken van ziekenhuispersoneel naast het bed |  |  |  |  |  |
| Piepende onderdelen van bed of apparatuur |  |  |  |  |  |
| Geluiden van patiënten zoals hoesten, snurken, kokhalzen of kreunen. |  |  |  |  |  |
| Televisie |  |  |  |  |  |
| Toilet doorspoelen |  |  |  |  |  |
| Apparatuur die wordt gebruikt voor patiënten zoals zuig- en of beademingsmachine |  |  |  |  |  |
| Vallende voorwerpen zoals po’s en patiënten mappen |  |  |  |  |  |
| Telefoons |  |  |  |  |  |
| Medicatie en linnenkarren |  |  |  |  |  |
| Radio |  |  |  |  |  |
| Dienbladen en bestek |  |  |  |  |  |

Wilt u nog iets kwijt over uw slaapkwaliteit tijdens opname in het ziekenhuis?

……………………………………………………………………………………………………………………………………………………………………………………………………………………………………………………………………………………………………………………………………………………………………………………………………………………………………………………………………………………………………………………………………………………………………………………………………………………………………………………………………………………………………………………………………………………………………………………………
