# Supplementary material for: A cross-sectional validation study of the verran and snyder-halpern sleep scale and the factors influencing sleep questionnaire in Dutch hospitals
Source: PLoS One. 2026 Sep 1;21(9):e0354633. doi: 10.1371/journal.pone.0354633 (PMC13533380; doi:10.1371/journal.pone.0354633)
Supplement: Supplementary file 3 [file pone.0354633.s003.docx]

**Supplemental file 3: Translation English- Dutch VSH and FISQ**

| **VSH** |  |
| --- | --- |
| **English** | **Dutch** |
| 1. Before you were admitted to hospital, did you have any regular difficulty with sleeping? | Had u, voordat u in het ziekenhuis werd opgenomen, al regelmatig problemen met slapen? |
| 2. Have you ever been diagnosed with a sleep problem? | Is er ooit een slaapprobleem bij u vastgesteld? |
| 3a. Have you taken any medications to help with sleep in the past year? | Heeft u in het afgelopen jaar medicatie ingenomen om te helpen bij het slapen? |
| 3b. If you answered “YES” for 3a, how many nights on average will you take a medication sleep aid at home? | Als u JA lap geantwoord op vraag 3a, hoeveel nachten neemt u gemiddeld thuis slaapmedicatie? |
| 4. Have you done any shift-based work in the past year? | Heeft u in het afgelopen jaar in ploegendiensten gewerkt? |
| 5a. How does the quality of your sleep in hospital compare with out of hospital (e.g. home)? | Hoe is uw slaapkwaliteit in het ziekenhuis in vergelijking met elders (bijvoorbeeld thuis)? |
| 5b. If you answered “ BETTER” or “WORSE” for #5a, why do you think your sleep in hospital is of better or worse quality? | Wanneer u BETER of SLECHTER lap geantwoord bij vraag 5a, waarom denkt u dat de kwaliteit van uw lap in het ziekenhuis beter of slechter is? |
| 6a. How many hours on average do you sleep at home during night-time hours? | Hoeveel uur slaapt u gemiddeld ’s nachts thuis? |
| 6b. How many hours on average do you sleep in hospital during night-time hours? | Hoeveel uur slaapt u gemiddeld per nacht in het ziekenhuis? |
| 7a. How many hours on average do you sleep at home during daytime hours (i.e. napping)? | Hoeveel uur slaapt u gemiddeld overdag thuis (bijvoorbeeld dutjes)? |
| 7b. How many hours on average do you sleep in hospital during daytime hours (i.e. napping)? | Hoeveel uur slaapt u gemiddeld overdag in het ziekenhuis (bijvoorbeeld dutjes)? |
| 8. What time did you first fall asleep last night? | Hoe laat viel u voor het eerst in slaap gisteravond? |
| 9. What time did you wake up this morning? | Hoe laat werd u vanmorgen wakker? |
| 10. Was your sleep continuous?  For the next set of questions, answer by placing an X on the answer line. Place your X anywhere along the line that you feel best describes your last night’s sleep.  The following is an example of the questions you are about to answer: | Sliep u aan een stuk door?  *Hierna volgen een aantal voorbeeldvragen:*  *Zet een X op de plaats die het beste aangeeft hoe u afgelopen nacht geslapen heeft.*  *Onderstaande voorbeelden geven aan hoe u de vragen dient te beantwoorden* |
| **Example A:**  During my sleep last night, I:  Did not awaken 0–––––––––– 10  Was awake 10 hours  If you were awake for 10+ hours, you would put your X at the end of the line, next to the words “Was awake 10 hours.” | **Voorbeeld A***: Tijdens mijn slaap van de afgelopen nacht:*  *Werd ik niet wakker 0------------10 Was ik 10 uur wakker*  *Als u meer dan 10 uur wakker was, dan zet u uw X aan het eind van de lijn, naast de woorden “Was ik 10 uur wakker.”* |
| **Example B:**  During my sleep last night, I:  Did not awaken 0––––––––––10 Was awake 10 hours  If you were awake for 5 hours, you would put your X in the middle of the line. | **Voorbeeld B:** *Tijdens mijn slaap van de afgelopen nacht:*  *Werd ik niet wakker 0-----10 Was ik 10 uur wakker*  *Als u 5 uur wakker was, dan zet u uw X in het midden van de lijn.* |
| **Example C:**  During my sleep last night, I:  Did not awaken 0–––––––––– 10 Was awake 10 hours  If you were asleep for the entire night, you would put your X at the start of the line. | **Voorbeeld C:** *Tijdens mijn slaap van de afgelopen nacht:*  *Werd ik niet wakker 0----10 Was ik 10 uur wakker*  *Als u de hele nacht geslapen heeft, dan zet u uw X aan het begin van de lijn.* |
| Place your X anywhere on the answer line where it best represents your sleep last night: | Plaats de X op de plek op de antwoordlijn die volgens u het beste weergeeft hoe u de afgelopen nacht heeft geslapen. |
| 11. Did not awake 0––––10 Was awake 10 hours | Ik werd niet wakker 0---10 was ik 10 uur wakker |
| 12. Had no sleep 0–––10 Had 10 hours sleep | Heb ik niet geslapen 0-----10 Heb ik 10 uur geslapen |
| 13. No sleep during the 0––10 Slept 10 hours during day yesterday | Gisteren overdag heb ik geslapen 0---10 Gisteren heb ik 10 uur overdag geslapen |
| 14. Did not sleep 0––10 Slept off and on  yesterday morning | Gisteren overdag heb ik geslapen 0-10 gisteren heb ik 10 uur overdag geslapen |
| 15. Did not sleep 0–– 10  Slept off and on  yesterday evening | Ik heb niet geslapen gisteravond 0---10 Ik heb gisteravond zo nu en dan geslapen |
| 16. Fell asleep 0–––––––––– 10 Did not fall asleep immediately | Gisteravond viel ik onmiddellijk in slaap 0---10 Gisteravond viel ik niet in slaap |
| 17. Slept lightly 0–––––– 10 Slept deeply | Licht geslapen 0-----10 Diep geslapen |
| 18. Had no trouble with 0––––––10 Had a lot of trouble disrupted sleep | Geen last van verstoorde slaap 0-----10 Veel last van verstoorde slaap |
| 19. Didn’t wake at all 0––10 Was awake off and on all night | Werd niet wakker 0------10 Was ik afwisselend wakker gedurende de hele nacht |
| 20. Had no trouble falling asleep 0––––––10 Had a lot of trouble falling asleep | Had geen problemen met in slaap komen 0------10 Had veel problemen met in slaap komen |
| 21. Didn’t move 0–– 10 Tossed all night | Niet bewogen 0-----10 De hele nacht liggen draaien |
| 22. Awoke refreshed 0–– 10 Awoke exhausted | Vanochtend werd ik uitgerust wakker 0-------10 Werd ik uitgeput wakker |
| 23. After morning awakening, dozed off 0 –––10 After morning awakening, stayed awake | Na het ontwaken vanochtend ben ik wakker gebleven 0------10 Na het ontwaken vanochtend ben ik weer in slaap gedommeld |
| 24. Had a bad night’s sleep 0––––10 Had a good night’s sleep | Een slechte nachtrust gehad 0------10 Een goede nachtrust gehad |
| 25. Had enough sleep 0–––10 Did not have enough | Had voldoende slaap 0-------10 Niet voldoende slaap |
| **FISQ** | |
| **English** | **Dutch** |
| Pain | Pijn |
| Comfort bed and environment | Comfort van bed en omgeving |
| Air conditioning, heating or ventilation system | Airconditioning, verwarming of ventilatiesysteem |
| Anxiety | Angst |
| Nurses’ interruptions | Verstoring door verpleegkundigen |
| Lights | Verlichting |
| Doors opening, closing, slamming | Deuren die geopend, gesloten of dichtgeslagen worden |
| Socializing at the nurses’ station | Gesprekken in personeelsruimte |
| Alarms on equipment | Alarmen op apparatuur |
| Paging system | Oproepsysteem |
| Loud talking in the hallway at night | ‘s nachts luid gepraat in de gang |
| Visitors | Bezoekers |
| Loud talking during day | Luid gepraat overdag |
| Cleaning equipment, such as vacuum cleaners | Schoonmaak apparatuur zoals stofzuiger |
| Footsteps | Voetstappen |
| Handwashing at nearby sink | Handen wassen bij een wasbak in de buurt |
| Intercom, call lights | Intercom |
| Conversations between hospital personnel at the bedside | Gesprekken van ziekenhuispersoneel naast het bed |
| Squeaking parts on beds or equipment | Piepende onderdelen van bed of apparatuur |
| Patient sounds such as coughing, snoring, gagging, moaning | Geluiden van patiënten zoals hoesten, snurken, kokhalzen en kreunen |
| Television | Televisie |
| Toilets flushing | Toilet doorspoelen |
| Equipment used for patients such as suction and/or breathing machines | Apparatuur die wordt gebruikt voor patiënten zoals zuig- en of beademingsmachine |
| Falling objects such as pans, patients charts | Vallende voorwerpen zoals po’s en patiënten mappen |
| Telephones | Telefoons |
| Medicine and linen carts | Medicatie en linnenkarren |
| Radio | Radio |
| Meal trays and eating utensils | Dienbladen en bestek |
