# Supplementary material for: A cross-sectional validation study of the verran and snyder-halpern sleep scale and the factors influencing sleep questionnaire in Dutch hospitals
Source: PLoS One. 2026 Sep 1;21(9):e0354633. doi: 10.1371/journal.pone.0354633 (PMC13533380; doi:10.1371/journal.pone.0354633)
Supplement: Supplementary file 4 [file pone.0354633.s004.docx]

**Supplemental file 4: Residual item covariances added to the VSH model**

VSH_fallingasleep ~~ VSH_problemfallingasleep

VSH_nightssleep ~~ VSH_sleepdepth

VSH_disturbedsleep ~~ VSH_notalternatelyawake

VSH_sleepdepth ~~ VSH_notalternatelyawake

VSH_didnotsleepwell ~~ VSH_nightsleep

VSH_didnotsleepwell ~~ VSH_sleepdepth

VSH_wellnotawake ~~ VSH_disturbedsleep

VSH_wellnotawake ~~ VSH_notalternatelyawake
