# Supplementary material for: A cross-sectional validation study of the verran and snyder-halpern sleep scale and the factors influencing sleep questionnaire in Dutch hospitals
Source: PLoS One. 2026 Sep 1;21(9):e0354633. doi: 10.1371/journal.pone.0354633 (PMC13533380; doi:10.1371/journal.pone.0354633)
Supplement: Supplementary file 5 [file pone.0354633.s005.docx]

**Supplemental file 5**

**Table 1:** Factor loading and reliability estimates for the three-factor VSH model.

| **Questions** | **Item number** | **Sleep  Effectiveness** | **Sleep  Disturbance** | **Sleep  Supplementation** |
| --- | --- | --- | --- | --- |
| Had enough sleep – Did not have enough | VSH_25 | 0.787 | - | - |
| Awoke refreshed – Awoke exhausted | VSH_22 | 0.752 | - | - |
| Had a bad night’s sleep – Had a good night’s sleep | VSH_24 | 0.704 | - | - |
| Had no sleep – Had 10 hours sleep | VSH_12 | 0.275 | - | - |
| Did not awake - Was awake 10 hours | VSH_11 | - | 0.696 | - |
| Fell asleep – Did not fall asleep immediately | VSH_16 | - | 0.548 | - |
| Slept lightly – Slept deeply | VSH_17 | - | 0.487 | - |
| Had no trouble with – Had a lot of trouble disrupted sleep | VSH_18 | - | 0.714 | - |
| Didn’t wake at all – Was wake off and on all night | VSH_19 | - | 0.662 | - |
| Had no trouble falling asleep – Had a lot of trouble falling asleep | VSH_20 | - | 0.756 | - |
| Didn’t move – Tossed all night | VSH_21 | - | 0.637 | - |
| No sleep during – Slept 10 hours during the day yesterday | VSH_13 | - | - | 0.560 |
| Did not sleep - Slept off and on yesterday morning | VSH_14 | - | - | 0.750 |
| Did not sleep – Slept off and on yesterday evening | VSH_15 | - | - | 0.472 |
| After morning awakenings, dozed off – After morning awakening, stayed awake | VSH_23 | - | - | 0.378 |
|  | Reliability (McDonald’s Omega) | 0.74 | 0.80 | 0.63 |

**Table 2:** Factor loading estimates for the one factor FISQ model.

| **Item name** | **Item number** | **Median (IQR)** | **Standardized loading** |
| --- | --- | --- | --- |
| **Pain** | FISQ_1 | **1 (0-3)** | 0.307 |
| **Comfort of bed and surrounding** | FISQ_2 | **2 (0-3)** | 0.344 |
| Anxiety | FISQ_4 | 0 (0-0) | 0.380 |
| Nurses’ interruptions | FISQ_5 | 0 (0-1) | 0.605 |
| Trolleys in hallway | FISQ_ 26 | 0 (0-0) | 0.766 |
| Meal trays | FISQ_28 | 0 (0-0) | 0.776 |
| Lights | FISQ_6 | 0 (0-1) | 0.592 |
| Intercom | FISQ_17 | 0 (0-0) | 0.875 |
| Doors opening/closing/slamming | FISQ_7 | 0 (0-1) | 0.752 |
| Talking at nurses’ station | FISQ_8 | 0 (0-0) | 0.750 |
| Visitors | FISQ_12 | 0 (0-0) | 0.763 |
| Cleaning equipment | FISQ_14 | 0 (0-0) | 0.784 |
| Footsteps | FISQ_15 | 0 (0-1) | 0.642 |
| Handwashing | FISQ_16 | 0 (0-0) | 0.757 |
| **Ventilation systems** | FISQ_3 | **1 (0-2)** | 0.276 |
| **Equipment alarms** | FISQ_9 | **1 (0-1)** | 0.650 |
| Paging | FISQ_10 | 0 (0-0) | 0.734 |
| Talking in hallway at night | FISQ_11 | 0 (0-0) | 0.756 |
| Talking in hallway during day | FISQ_13 | 0 (0-1) | 0.616 |
| Staff talking at bedside | FISQ_18 | 0 (0-1) | 0.754 |
| Squeaky equipment | FISQ_19 | 0 (0-1) | 0.693 |
| **Patient sounds** | FISQ_20 | **1 (0-2)** | 0.663 |
| Television | FISQ_21 | 0 (0-0) | 0.795 |
| Procedures on other patients | FISQ_23 | 0 (0-0) | 0.703 |
| Falling objects | FISQ_24 | 0 (0-0) | 0.746 |
| Telephones | FISQ_25 | 0 (0-0) | 0.778 |
| Radio | FISQ_27 | 0 (0-0) | 0.850 |

Note*: Omega total reliability estimate = 0.93*
